# miR‐199a‐3p and miR‐214‐3p improve the overall survival prediction of muscle‐invasive bladder cancer patients after radical cystectomy

**DOI:** 10.1002/cam4.1161

**Published:** 2017-09-06

**Authors:** Thorsten H. Ecke, Katja Stier, Sabine Weickmann, Zhongwei Zhao, Laura Buckendahl, Carsten Stephan, Ergin Kilic, Klaus Jung

**Affiliations:** ^1^ Department of Urology HELIOS Hospital Bad Saarow Germany; ^2^ Department of Urology Campus Benjamin Franklin University Hospital Charité Germany; ^3^ Department of Urology Campus Charité Mitte University Hospital Charité Germany; ^4^ Berlin Institute for Urologic Research Berlin Germany; ^5^ Institute of Pathology University Hospital Charité Germany

**Keywords:** Biomarker, microRNAs, multivariate analysis, muscle‐invasive bladder cancer, overall survival, prognosis

## Abstract

To improve the clinical decision‐making regarding further treatment management and follow‐up scheduling for patients with muscle‐invasive bladder cancer (MIBC) after radical cystectomy (RC), a better prediction accuracy of prognosis for these patients is urgently needed. The objective of this study was to evaluate the validity of differentially expressed microRNAs (miRNAs) based on a previous study as prognostic markers for overall survival (OS) after RC in models combined with clinicopathological data. The expression of six miRNAs (miR‐100‐5p, miR‐130b‐3p, miR‐141‐3p, miR‐199a‐3p, miR‐205‐5p, and miR‐214‐3p) was measured by RT‐qPCR in formalin‐fixed, paraffin‐embedded tissue samples from 156 MIBC patients who received RC in three urological centers. Samples from 2000 to 2013 were used according to their tissue availability, with follow‐up until June 2016. The patient cohort was randomly divided into a training (*n* = 100) and test set (*n* = 56). Seventy‐three samples from adjacent normal tissue were used as controls. Kaplan–Meier, univariate and multivariate Cox regression, and decision curve analyses were carried out to assess the association of clinicopathological variables and miRNAs to OS. Both increased (miR‐130b‐3p and miR‐141‐3p) and reduced (miR‐100‐5p, miR‐199a‐3p, and miR‐214‐3p) miRNA expressions were found in MIBC samples in comparison to nonmalignant tissue samples (*P* < 0.0001). miR‐199a‐3p and miR‐214‐3p were independent markers of OS in Cox regression models with the significant clinicopathological variables age, tumor status, and lymph node status. The prediction model with the clinicopathological variables was improved by these two miRNAs in both sets. The predictive benefit was confirmed by decision curve analysis. In conclusion, the inclusion of both miRNAs into models based on clinical data for the outcome prediction of MIBC patients after RC could be a valuable approach to improve prognostic accuracy.

## Introduction

Bladder cancer is the fifth most frequent cancer in Europe. In 2012, its incidence and annual mortality rate were estimated to 151,200 and 51,400 cases, respectively 1. Approximately 30% of these patients suffered from muscle‐invasive bladder cancer (MIBC) at the time of initial diagnosis 2. Radical cystectomy (RC) is the gold standard to treat these patients. In contrast to patients with nonmuscle‐invasive bladder cancer (NMIBC), MIBC patients are subject to a high risk of relapse following RC and cancer‐related death.

In order to remedy this unsatisfactory situation, serious efforts have recently focused on new therapeutic strategies regarding the application of neoadjuvant and adjuvant chemotherapies 3. A better risk assessment of patients has been recommended by developing novel predictive/prognostic models 4. In clinical practice, the therapeutic management of these patients has so far been performed almost exclusively on the basis of clinical data and classical pathological TNM criteria but with few reliable results 4. There is the hope that the identification of new molecular tissue biomarkers could help to stratify risk groups and determine which patients benefit from adjuvant strategies after surgery 5. While the results of numerous studies using immunohistochemical biomarkers have been rather disappointing regarding their clinical utilization 6, recent reports on gene‐based approaches 5, 7, 8, including using microRNAs (miRNAs) as a new class of mRNA regulators seem to be much more promising 9, 10, 11, 12, 13, 14, 15, 16, 17.

In a previous study, we identified numerous new differentially expressed miRNAs in fresh‐frozen bladder cancer tissue samples in comparison to normal adjacent tissue 18. It was therefore the objective of this study to evaluate from these 15 previously described miRNAs the usefulness of the most promising miRNAs regarding their potential predictive ability of overall survival (OS) and assess their benefit in comparison to conventional clinicopathological variables in MIBC patients after RC. Criteria for the selection of these miRNAs in this study were determinations in a well measurable Cq range and at least twofold median expression differences between the nonmalignant and MIBC tissue samples to allow robust measurements as described before 18. According to these criteria, both three up‐regulated (miR‐130b‐3p, miR‐141‐3p, and miR‐205‐5p) and three down‐regulated (miR‐100‐5p, miR‐199a‐3p, and miR‐214‐3p) miRNAs were included in this study. To simulate the conditions of clinical practice formalin‐fixed, paraffin‐embedded (FFPE) tissue samples from three urological centers were analyzed corresponding to a multi‐center study.

## Materials and Methods

### Patients and tissue samples

This study included 156 MIBC patients without any neoadjuvant therapy who underwent RC at three urological centers from 2000 to 2013. Sixty‐four patients (41%) received adjuvant cisplatin‐based chemotherapy after RC (Table 1). Follow‐up data collected until June 2016 were based on medical records and telephone contact with the patients’ urologists and patients or family members and death certificates. The study was approved by the Hospital Ethics Committee (EA1/153/07; EA1/134/12) in accordance with the Declaration of Helsinki. The new STARD and REMARK guidelines were considered 19, 20.

**Table 1:** Clinicopathological characteristics of the study groups

Variable	Controls^a^ (*n* = 73)	All MIBC patients (*n* = 156)	*P* b	Training set (*n* = 100)	Test set (*n* = 56)	*P* b
Age [year, median (range)]	69 (44–81)	69 (37–82)	0.689	69 (37‐82)	68 (45–81)	0.283
Gender (*n*; %)			0.401			0.241
Female	7 (10)	23 (15)		12 (12)	11 (20)	
Male	66 (90)	133 (85)		88 (88)	45 (80)	
pT status (*n*; %)						
pT2		47 (30)		32	15	0.759
pT3		78 (50)		48	30	
pT4		31 (20)		20	11	
Grade (*n*; %)						
G2		14 (9)		10	4	0.463
G3		140 (90)		88	52	
G4		2 (1)		2	‐	
pN status (*n*; %)						
pN0/Nx		102 (65)		69	33	0.222
pN1		54 (35)		31	23	
Adjuvant therapy (*n*; %)						
Yes		64 (41)		43	21	0.611
No		92 (59)		57	35	
Sample source (*n*; %)^c^						
Center 1	51 (70)	101 (65)	0.486	62	39	0.598
Center 2	7 (10)	24 (15)		16	8	
Center 3	15 (20)	31 (20)		22	9	
Follow‐up after surgery						
Time [month; median (range)]		28 (1–180)		34 (1–180)	23 (1–163)	0.503
Death events (n; %)		99 (63)		67 (67)	32 (57)	0.230
Survival time [month, median (95% CI)]		34 (24–49)		35 (24–49)	26 (16–106)	0.589

CI, confidence interval; G, histopathological grade; MIBC, muscle‐invasive bladder cancer; pN, lymph node status; pT, pathological tumor classification.

a Controls refer to nonmalignant tissue samples obtained from MIBC patients as described in Materials and Methods.

b Statistical tests: Mann–Whitney *U* test; Chi‐square or Fisher's exact test, and log‐rank test, Kaplan–Meier analysis.

c Center 1: Campus Benjamin Franklin, University Hospital Charité; Center 2: Campus Mitte, University Hospital Charité; Center 3: Helios Clinical Center, Bad Saarow.

The study was carried out on FFPE tissue specimens selected according to their tissue availability and follow‐up data (Table 1). All samples were finally reviewed by an expert uropathologist (EK) according to the criteria of the International Union Against Cancer and the World Health Organization/International Society of Urological Pathology, as previously reported 18.

### RNA extraction and quantitative RT‐PCR of miRNAs

Previously described analytical procedures were applied 18, 21. Hematoxylin/eosin staining was used to identify tumor areas with >80% tumor cells and nonmalignant areas. The areas of interest were punch‐biopsied with a 1‐mm needle device and extracted with the miRNeasy FFPE Kit with an additional DNase I digestion step (Qiagen, Hilden, Germany). RT‐qPCR measurements and normalizations were performed for the quantification of the miRNAs with TaqMan miRNA assays (Applied Biosystems, Foster City; Table S1) on a Light Cycler 480 Instrument (Roche Diagnostics, Mannheim, Germany) as previously described including the related documentation with regard to the specific items of the MIQE guidelines 18, 21, 22.

### Statistical analysis

QBasePLUS, v.3.0 software (Biogazelle, Zwijnaarde, Belgium) was used for the analysis of RT‐qPCR data. Statistical analyses were performed with SPSS 23 (SPSS Inc., Chicago) and MedCalc 16.8.4 (MedCalc Software, Ostend, Belgium). Nonparametric statistical tests (Spearman rank correlation, Mann‐Whitney *U* test, Kruskal–Wallis test) were used. The discriminating capacity of miRNAs was assessed by receiver operating characteristics (ROC) analysis and binary logistic regression. Kaplan–Meier and Cox regression analyses were performed for OS. Decision curve analysis and time‐dependent ROC analysis using the statistical approach of cumulative case/dynamic control and incident case/dynamic control ROC analysis were used to assess the predictive benefit and accuracy of the examined markers 23, 24. The SPSS bias‐corrected and accelerated bootstrap method was used for the internal validation using the patient cohort by splitting at random into a training (*n* = 100) and test set (*n* = 56). *P*‐values <0.05 (two‐tailed) were considered statistically significant.

## Results

### Study design and patient characteristics

This retrospective study from three urologic departments included 156 MIBC patients that were randomly divided into a training (*n* = 100) and test set (*n* = 56). For comparison purposes, 73 nonmalignant tissue samples available from these patients were used as controls. The characteristics of the study groups are summarized in Table 1. There were no statistically significant differences in age and sex between the controls and MIBC patients and between the training and test set with regard to the important clinicopathological variables (Table 1).

### Differential expression of microRNAs in bladder tissue of MIBC patients

**Figure 1:**
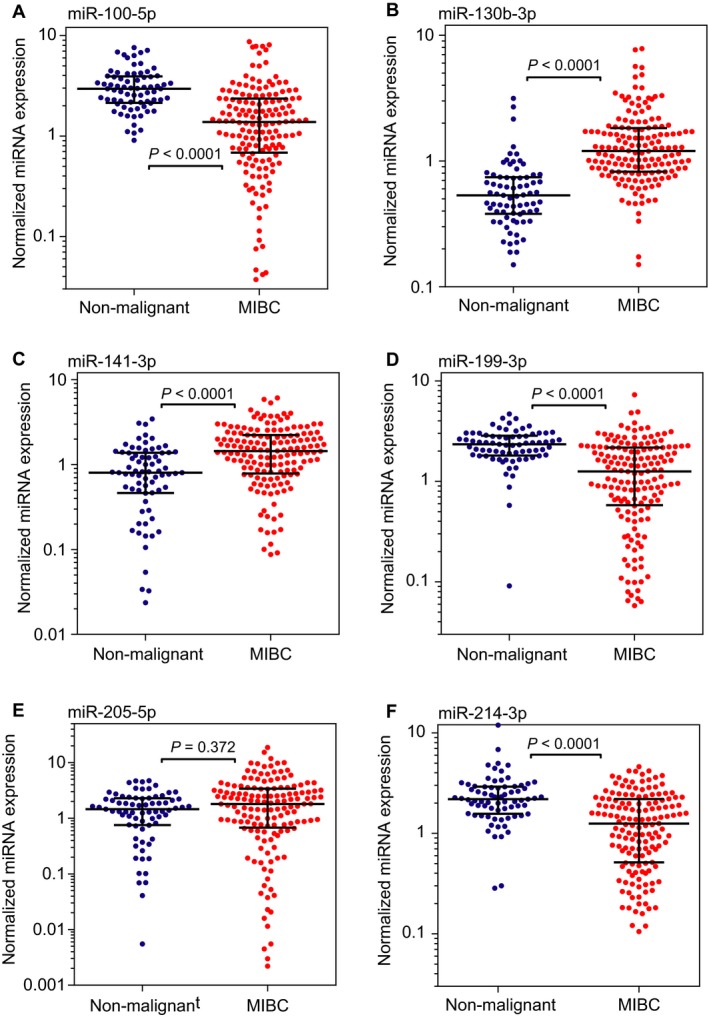
Differential expression of miRNAs (A: miR‐100‐5p; B: miR‐130‐3p; C: miR‐141‐3p; D: miR‐199‐3p; E: miR‐205‐5p; F: miR‐214‐3p) in nonmalignant bladder tissue (*n* = 73) and muscle‐invasive bladder cancer (MIBC) tissue samples (*n* = 156). Expression values were normalized against the reference miRNA signature (miR‐101‐3p, miR‐148b‐5p, miR‐125a‐5p, and miR‐151‐5p) as previously described 21. Medians and interquartile ranges are indicated with statistical significances calculated by the Mann–Whitney *U* test.

The expression data of the six miRNAs of interest (miR‐100‐5p, miR‐130b‐3p, miR‐141‐3p, miR‐199a‐3p, miR‐205‐5p, miR‐214‐3p) were not statistically different (*P* = 0.084–0.792) between the three centers, so merged data could be evaluated. Figure 1 shows that the miRNA expressions differed with high statistical significance (*P* < 0.0001) between nonmalignant and MIBC samples except miR‐205‐5p. Both increased (miR‐130b‐3p, miR‐141‐3p) and reduced levels (miR‐100‐5p, miR‐199a‐3p, miR‐214‐3p) were found in MIBC samples in comparison to nonmalignant tissue samples (*P* < 0.0001). The differential miRNA expressions corresponded to the capacity of the miRNAs to discriminate between nonmalignant and MIBC tissue with miR‐130b‐3p as the best discriminator (Table S2 and Fig. S1A and S1B).

### Correlation of miRNA expressions to clinicopathological variables and among each other

Correlations were not observed between all miRNAs and sex or age in nonmalignant and tumor samples (*r*_s_ = 0.108–0.001, *P*‐values of 0.180–0.915). The clinicopathological variables pT status, histopathological grade, and lymph node status were also not significantly associated with all miRNAs (*r*_s_ = 0.113–0.004, *P‐*values of 0.160–0.957) except for miR‐130b‐3p and miR‐205‐5p to grade (*r*_s_ = 0.236 to −0.197, *P*‐values of 0.003 and 0.014). These missing associations of miRNAs to clinicopathological variables corresponded to results obtained by the Kruskal–Wallis (pT status, histological grade) and Mann–Whitney test (lymph node status). Different correlation coefficients between miRNAs were observed between nonmalignant and MIBC tissue (Table S3).

### Prognostic potential of miRNAs predicting postcystectomy overall survival of MIBC patients

The differential expression and the different correlations between the examined miRNAs indicate their potential as prognostic markers. To substantiate the relationship between the clinical outcome of patients and the clinicopathological variables as well as miRNAs, OS was analyzed as the primary clinical endpoint since reliable information about cancer death was not available in all cases. OS was defined as the time from the date of RC until the time of death or the last follow‐up.

Kaplan–Meier survival analyses of clinicopathological variables showed a decreased OS depending on the lymph node metastasis, increased pT status, and age, but OS was not associated with adjuvant chemotherapy (Fig. S2). These initial data proved the representativeness of the study cohort to evaluate the impact of miRNAs regarding their usefulness as prognostic markers. For the internal validation of data, the prognostic performance of all clinicopathological variables and the six miRNAs was calculated using the training and test set (Table 2). The variables with *P*‐values <0.10 in univariable analyses for clinicopathological factors (age, pT status, pN status) and for the two miRNAs miR‐199a‐3p, miR‐214‐3p were used to build up two models, a full model with all five variables and a reduced model after a backward elimination approach (Table 2). This threshold of *P*‐value <0.10 was selected to avoid a possible false negative decision (Type II error) for a potential relevant parameter using a low alpha value in this first step of model building based on univariable Cox regression analysis. Both miRNAs also remained as statistically independent variables in the model after backward elimination. In addition, the training and test set gave closely corresponding results, indicating that an overfitting bias of results can be largely excluded. It is of a special interest that the above mentioned miR‐130b‐3p as the best discriminator between malignant and nonmalignant bladder tissue failed to provide any prognostic information (Table 2).

**Table 2:** Cox proportional hazard regression analyses of clinicopathological factors and miRNAs for predicting overall survival in MIBC patients after radical cystectomy in training and test set^a^

Variable^b^	Training set (*n* = 100)		Test set (*n* = 56)	
	HR (95% CI)	*P‐*value	HR (95% CI)	*P‐*value
Univariable analysis				
Age (<69/≥69 years)	1.56 (0.94–2.53)	0.087	1.98 (0.99–3.97)	0.055
Sex (female/male)	1.61 (0.73–3.53)	0.283	1.20 (0.45–3.62)	0.659
pT status (pT2,3,4)	1.46 (1.04–2.04)	0.029	1.64 (0.83–3.44)	0.098
Grade (G2/G3‐4)	0.96 (0.46–2.01)	0.904	1.50 (0.29–23.9)	0.529
pN status (N0,Nx/N1)	2.01 (1.23–3.28)	0.005	1.96 (0.94–4.65)	0.053
Adjuvant therapy	1.38 (0.85–2.22)	0.193	0.89 (0.44–1.79)	0.741
miR‐100‐5p	0.99 (0.87–1.23)	0.853	0.93 (0.60–1.12)	0.680
miR‐130b‐3p	0.99 (0.77–1.27)	0.918	1.02 (0.53–1.85)	0.643
miR‐141‐3p	0.93 (0.70–1.25)	0.633	0.76 (0.39–1.24)	0.306
miR‐199a‐3p	0.53 (0.30–0.91)	0.023	0.49 (0.18–0.96)	0.042
miR‐205‐5p	1.02 (0.92–1.14)	0.720	1.16 (0.81–1.95)	0.302
miR‐214‐3p	1.80 (1.12–2.89)	0.015	2.96 (1.29–6.75)	0.005
Multivariable analysis, full model^c^				
Age	1.49 (0.88–2.51)	0.137	1.65 (0.77–3.53)	0.255
pT status	1.45 (1.01–2.07)	0.045	1.85 (0.99–3.43)	0.053
pN status	1.62 (0.97–2.71)	0.064	2.26 (1.01–5.04)	0.046
miR‐199a‐3p	0.57 (0.32–1.02)	0.058	0.32 (0.11–0.94)	0.039
miR‐214‐3p	1.79 (1.12–2.85)	0.015	3.30 (1.11–9.77)	0.031
Multivariable analysis, backward elimination^d^				
pT status	1.42 (1.01–1.98)	0.042	1.81 (0.96–3.39)	0.065
pN status	1.67 (1.01–2.77)	0.052	2.32 (1.06–5.09)	0.035
miR‐199a‐3p	0.53 (0.27–0.89)	0.026	0.35 (0.13–0.91)	0.032
miR‐214‐3p	1.88 (1.21–3.51)	0.005	3.29 (1.24–8.74)	0.017

CI, confidence interval; G, histopathological grading; HR, hazard ratio; MIBC, muscle‐invasive bladder cancer; miR, microRNA; pT, pathological tumor classification; pN, lymph nodal status.

a The training set included 100 and the test set 56 of MIBC patients randomly selected from the cohort characterized in Table 1.

b Calculations were performed by bootstrapping (2000 resamples) for clinical variables using categorized data as indicated in brackets and for miRNAs using normalized continuous expression values.

c The multivariable analysis included all variables with *P‐*values <0.10 obtained in the univariable analysis to avoid a type II error in the first step of model building.

d The backward multivariable analysis (*P* = 0.05 for entry; *P* = 0.10 for removal) was based on the five variables used in the full multivariable analysis. The 95% CI of the hazard ratios and the *P*‐values of the final model were obtained after bootstrapping (2000 resamples).

**Figure 2:**
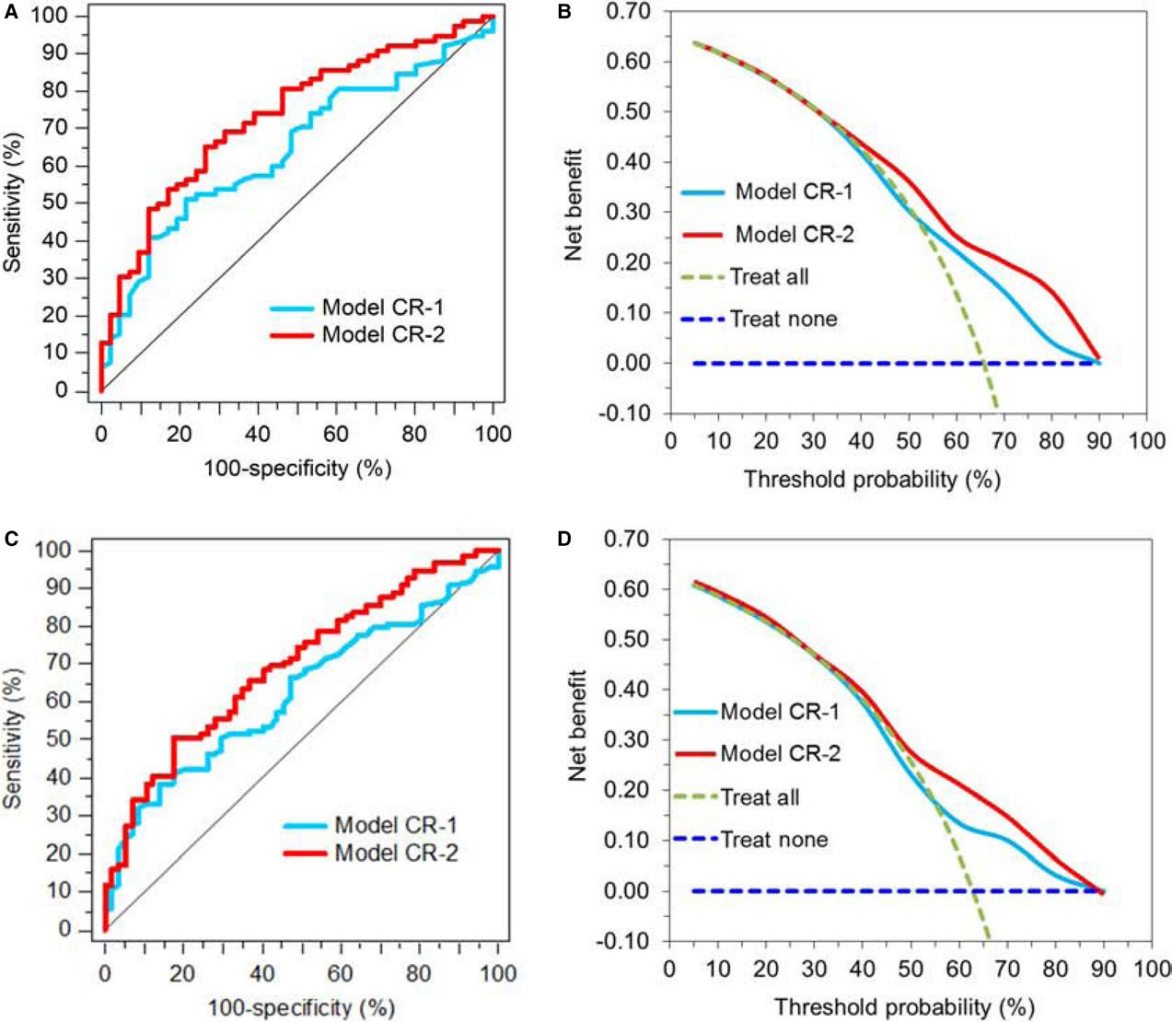
Improved survival predictive accuracy by including miR‐199a‐3p and miR‐214‐3p (Model CR‐2) in a model with only clinicopathological variables age, pT status, and lymph node metastasis (Model CR‐1). Areas under the time‐dependent ROC curve (AUC) of the two models were calculated based on (A) a cumulative case (*n* = 78)/dynamic control (*n* = 41) approach at 36 months after surgery as well as on (C) an incident case (*n* = 99)/dynamic control (*n* = 57) approach 24. AUCs of Model CR‐2 showed statistically significant higher values in both approaches in comparison with Model CR‐1 [Model CR‐2 vs. Model CR‐1 in (A) with 0.735 (0.624–0.827) vs. 0.645 (0.545–0.746), *P* = 0.031 and in (C) with 0.709 (0.637–0.796) vs. 0.622 (0.534–0.716), *P* = 0.011]. Curves in the decision curve analysis confirmed (B and D) the benefit of including the two miRNAs in the model based only on clinicopathological variables.

To confirm the prognostic impact of miR‐199‐3p and miR‐214‐3p, *C*‐statistics were performed in a model with the three above‐mentioned relevant clinicopathological variables alone (Model CR‐1) in comparison to a model (Model CR‐2) that also included the relevant miR‐199a‐3p and miR‐214‐3p (Table 2). Based on the median survival time of about 3 years in the entire cohort (Table 1), the AUCs in cumulative case/dynamic and incident case/dynamic control ROC analyses were calculated (Fig. 2A and C). Both approaches resulted in statistically significant higher AUC values in the miRNA‐enriched model in comparison to the model with clinicopathological variables alone (AUC of 0.735 vs. 0.645 and 0.709 vs. 0.622; Fig. 2A and C). The predictive benefit of the two miRNAs is also shown in the decision curve analyses (Fig. 2B and D). The curves of the model with miRNAs (Model CR‐2) are always above the curves of the model with only the clinicopathological variables (Model CR‐1).

## Discussion

In this retrospective study exclusively focused on the outcome OS, we continued to translate our conclusions drawn from previous miRNA studies in fresh‐frozen tissue samples into clinical practice using archived tissue FFPE samples from three centers 18, 21. This study design follows the concept of a discovery‐driven global strategy for biomarker search in translational research 25, 26. It is a hypothesis‐generating approach that considers the complex situation in clinical settings with regard to all possible variables which could influence the selected clinical end point. We previously postulated four development phases of biomarker assays for clinical practice, namely, first, the discovery and selection of potential biomarkers, second, the assay setup and performance control, third, the validation by clinical assessment, and finally the validation by clinical usability 27. According to this scheme, we would classify our present study into the third phase. The study results suggest that miR‐199a‐3p and miR‐214‐3p are helpful biomarkers to enhance the OS prediction in comparison with clinicopathological data alone in MIBC patients after RC and improve therefore further decision‐making both for clinicians and patients.

Numerous miRNA studies in bladder cancer identified histological grade and pT classification dependent miR expressions and proved typical miRNA alterations related to the two divergent pathways found in the development of NMIBC and MIBC (reviewed by Guancial et al. 28). As already noted in the introduction, only a few studies used miRNAs as prognostic and predictive biomarkers in bladder cancer patients but mostly in NMIBC patients 9, 10, 11, 12, 13, 14, 15, 16, 17. Different miRNAs were suggested as prognostic biomarkers for MIBC patients as combinations of four (let‐7c, miR‐125b‐1, miR193a, miR‐99a) 17, three (miR‐9, miR‐183, miR‐200b) 13, and two miRNAs (miR‐143, miR‐145) 15, but also as predictive markers for the response to cisplatin‐based adjuvant chemotherapy 11. However, none of these studies performed multivariable analyses or verified the examined miRNAs as independent markers.

In a recent study, Schubert et al. summarized the inconsistent data situation of miRNAs as prognostic markers in urological tumors 29. Different strategies to identify suitable prognostic miRNAs, the use of various analytical techniques, the insufficient sample size of study groups with lack of internal/external validations and multivariable analyses are reasons for the partially contradictory results if miRNAs have been applied as prognostic biomarkers in bladder cancer 29. Furthermore, beside the application of different clinical endpoints, studies have been also performed in cohorts including both MIBC and NMIBC patients without separate data evaluation or missing multivariable analysis 16, 30. Distinct molecular alterations including different miRNA profiles in noninvasive and invasive tumors may facilitate discrimination between both bladder cancer entities 9, 31, 32, but this aspect needs a special attention for the prognostic assessment of miRNAs. The different miRNA expression, partly characterized by an opposite expression between nonmalignant, NMIBC, and MIBC tissue as for example in the case of miR‐141 and miR‐205 18, could hamper a reliable prediction of the clinical outcome in multivariable Cox regression analyses in connection with clinicopathological factors. This possible bias was impressively illustrated by the example of miR‐214 33. This miRNA was shown to be an independent factor of recurrence‐free survival and OS in the MIBC cohort but failed if MIBC and NMIBC patients were analyzed as a combined group. To avoid all these possible errors, we studied only one bladder cancer entity, MIBC patients. We used FFPE samples from the practical point of view with a sufficient sample size and number of events for carrying out multivariable analyses, and performed a twofold internal validation approach by using a training and test set with bootstrapping calculation of continuous instead of categorized data according to the REMARK guidelines to achieve a high level of statistical power.

The up‐ and down‐regulated expression of the six selected miRNAs primarily found in fresh‐frozen MIBC versus nonmalignant tissue in our previous study 18 was confirmed in the FFPE samples in this study reaching at least by a median twofold change, except for miR‐205‐5p (Fig. 1). Moreover, three points should be emphasized in relation to assessment of outcome as the main aim of this study: the missing or only weak correlations of miRNAs with the clinicopathological risk factors, the different correlation coefficients among the miRNAs described in the Results, and the failure of miR‐130‐3p as the best miRNA discriminator between nonmalignant and malignant tissue to provide any prognostic information. The striking hallmark of the uncorrelated differential expression of miRNAs to known disease variables characterizes the miRNAs here examined as potential orthogonal biomarkers 34. This particular feature of orthogonal markers appears as a precondition that such markers can influence the clinical endpoint of OS independent of the conventional clinicopathological variables and must therefore be included as necessary independent variables in a corresponding prognostic model. The evidence of such an independent variable in multivariable analysis additionally promotes the discovery of novel downstream targets and their associated pathways 34. Our study proved that both miR‐199a‐3p and miR‐214‐3p remained such independent variables in multivariable Cox regression models (Table 2). The predictive accuracy of OS probability based only on the conventional clinicopathological variables could be improved by these two miRNAs. This clinical benefit was verified by the decision curve analysis and also by the time‐dependent ROC analysis if the two miRNAs were included into the model with only conventional clinicopathological variables (Fig. 2). The combined use of the clinicopathological variables and the expression data of the two microRNAs measured in tissue samples from cystectomy specimens offers the opportunity to develop a risk scoring system in prospective studies as result of the clinical usability of this approach. This would correspond to the final development phase of biomarker assay for clinical practice as suggested above 27. In contrast to miR‐199a‐3p and miR‐214‐3p, miR‐130b‐3p as the best discriminator between nonmalignant and cancer tissue did not offer any prognostic information. The prognostic information could also not be confirmed for miR‐141‐3p and miR‐205‐5p, which were previously shown only in Kaplan–Meier analyses, but not in multivariable Cox regression analyses 16, 18. The reason might be that both miRNAs show an opposite expression in NMIBC and MIBC and the mentioned studies were performed in combined cohorts of NMIBC and MIBC patients.

Up to now there are only few investigations concerning the role of miR‐199a‐3p and miR‐214‐3p with special focus on bladder cancerogenesis and its neoplastic progression. miR‐199a derives from two loci of the human genome, mir‐199a‐1 of chromosome 19 (cytogenetic location 19p13.2) and mir‐199a‐2 of chromosome 1 (cytogenetic location 1q24.3). These two loci encode mir‐199a, which generate the two mature miRNAs, miR‐199a‐3p and miR‐199a‐5p. Depending on the underlying malignancy, miR‐199a‐3p acts both as oncogene being up‐regulated like in gastric and colorectal cancer 35, 36 and as tumor suppressor being down‐regulated like in renal cell cancer, hepatocellular carcinoma, prostate cancer or bladder cancer 37, 38, 39. The down‐regulated expression of miR‐199a‐3p in bladder cancer was also described as tumor suppressive miRNA by other groups but not further evaluated as prognostic marker 31, 32, 40, 41. After we had finished our study, Sakaguchi et al. 42 recently showed that the expression of all four miR‐199 family members consisting of miR‐199b‐3p, and miR‐199b‐5p in addition to the two above‐mentioned miR‐199a forms, was down‐regulated in bladder cancer. The decreased expression of the miRNAs was found to be associated with a poor OS of patients shown in Kaplan–Meier analyses but it was not additionally assessed in multivariable Cox regression analyses 42. It was shown that these miRNAs act as tumor suppressors by targeting *ITGA3* (integrin subunit *α*3). However, the increased *ITGA3* mRNA expression was not significantly associated with the survival of patients 42. This phenomenon of missing link between a differentially expressed miRNA and its verified target gene with regard to a clinical endpoint is understandable, bearing in mind that miR‐199a targets numerous genes that could affect the prognosis of patients. For example, miR‐199a‐3p was validated as tumor suppressive miRNA in other cancers like prostate cancer targeting *AURKA* (aurora kinase A) 39, in renal cell cancer *GSK3B* (glycogen synthase kinase 3 beta) 37, and in hepatocellular carcinoma *HGF* (hepatocyte growth factor), *MMP2* (matrix metallopeptidase 2), *VEGFA* (vascular endothelial growth factor A), and its corresponding receptors 43. So far, these targets have not been examined in bladder cancer as potential points of action for miR‐199a‐3p. However, all these genes play a significant role in signaling pathways of angiogenesis, invasion, and metastasis in cancer as shown in the software DIANA‐mirPath (http://diana.imis.athena-innovation.gr/DianaTools) 44. Moreover, it is of particular interest that the second relevant prognostic mature miRNA in our study, miR‐214‐3p, derives from the stem loop sequence of mir‐214 that clusters with mir‐199a‐2 of the same chromosome locus 1q24.3. Similar to miR‐199a‐3p, miR‐214‐3p acts, depending on the cancer types, oncogenic in osteosarcoma and oral cancer 45, 46 but tumor suppressive in breast, cervical, esophageal, and bladder cancers 33, 47, 48, 49, 50, 51. Based on our previous expression study 18, Wang et al. 33 characterized the down‐regulated miR‐214‐3p as a suppressive miRNA. This miRNA functions as an independent factor of OS in MIBC patients, targeting the oncogene *PDRG1* (p53 and DNA damage regulated 1). *SLC34A2* (solute carrier family 34 member 2) and several genes of the epithelial‐mesenchymal transition and the *NGAL/MMP‐9* (lipocalin 2/metallopeptidase 9) pathways were also verified or predicted to be negatively regulated by miR‐214‐3p in the process of bladder cancer development 50, 51.

The possible biological significance of the co‐expression of the two down‐regulated miR‐199a and miR‐214 was shown in testicular germ cell tumor 52. A self‐regulatory network of the two microRNAs together with *PSMD10* (proteasome 26S subunit, non‐ATPase 10), *TP53* (tumor protein p53), and *DNTM1* (DNA methyltransferase 1) was identified whose dysfunction results in tumor progression. As mentioned above, these two microRNAs also act as regulators of numerous target genes that have been already validated so far in urinary bladder and other solid cancers (target characteristics are given in Table S4). Moreover, based on our data of prognostic relevance of miR‐199a‐3p and miR‐214‐3p, the view is supported that such a regulatory network or at least a cooperative action of the microRNAs as shown in other cancer examples 53 could also exist in bladder cancer. A detailed investigation of the functional role of the two miRNAs would be worthwhile but was beyond the scope of this study. On the other hand, we share the view of Burke 54 that the functional role of biomarker might be, in a certain sense, useful to explain the biological rationale of biomarker but is not decisive for its clinical usability. Under this translational aspect, the primary focus in the search for a truly reliable biomarker has to be directed more toward the clinical benefit of the potential new biomarker in comparison to the so far used procedures in clinical practice.

Limitations of the study are, despite our precautions for bias‐free analyses using the internal validation by a training and test sets with bootstrapping, its retrospective nature on overall and not cancer‐specific survival, the lack of external validation, and the biomarker‐focused aspect without elucidating the molecular mechanisms as argued above.

## Conclusions

In summary, our study identified miR‐199a‐3p and miR‐214‐3p as independent prognostic biomarkers for the prediction of OS in MIBC patients after RC. Their inclusion in prognostic models based on relevant clinicopathological risk factors improved the predictive accuracy. Such enhanced information of combined clinicopathological and molecular data could support clinicians in their decision‐making in the treatment management and follow‐up scheduling after RC. It could also help patients who wish to be kept fully informed about their further life expectancy to plan and manage their remaining lifetime. Thus, further efforts through complimentary multicenter prospective randomized studies would be worth to translate these biomarkers into clinical practice as suggested.

## Conflict of Interest

None declared.

## Supporting information


**Table S1.** Details of the TaqMan microRNA assays.
**Figure S1.** Discriminative capacity of miRNA models.
**Table S2.** Receiver‐operating characteristics analyses of miRNAs and their combinations with comments to Figure S1 and data in Table S2.
**Table S3.** Spearman correlation coefficients of miRNA‐pairs with comments to the data.
**Figure S2.** Kaplan–Meier analyses of overall survivals of muscle‐invasive bladder cancer patients after radical cystectomy in association with clinicopathological variables.
**Table S4.** Target genes of miR‐199a‐3p and miR‐214‐3p.
