# Supplementary material for: miR‐199a‐3p and miR‐214‐3p improve the overall survival prediction of muscle‐invasive bladder cancer patients after radical cystectomy
Source: Cancer Med. 2017 Sep 6;6(10):2252–62. doi: 10.1002/cam4.1161 (PMC5633587; doi:10.1002/cam4.1161)
Supplement: Supplementary file 1 — Table S1. Details of the TaqMan microRNA assays. Figure S1. Discriminative capacity of miRNA models. Table S2. Receiver‐operating characteristics analyses of miRNAs and their combinations with comments to Figure S1 and data in Table S2. Table S3. Spearman correlation coefficients of miRNA‐pairs with comments to the data. Figure S2. Kaplan–Meier analyses of overall survivals of muscle‐invasive bladder cancer patients after radical cystectomy in association with clinicopathological variables. Table S4. Target genes of miR‐199a‐3p and miR‐214‐3p. [file CAM4-6-2252-s001.docx]

**Supporting Information**

**for**

**miR-199a-3p and miR-214-3p improve the overall survival prediction of muscle-invasive bladder cancer patients after radical cystectomy**

Thorsten H. Ecke, Katja Stier, Sabine Weickmann, Zhongwei Zhao, Laura Buckendahl, Carsten Stephan, Ergin Kilic, Klaus Jung

**CONTENT**

[**Supplementary Table S1.** Details of the TaqMan microRNA assays - 2 -](#_Toc486956234)

[**Supplementary Figure S1.** Discriminative capacity of miRNA models - 3 -](#_Toc486956235)

[**Supplementary Table S2.**  ROC analyses of miRNAs and their combinations - 4 -](#_Toc486956236)

[Comments to Supplementary Figure S1 and Supplementary Table S2 - 4 -](#_Toc486956237)

[**Supplementary Table S3.**  Spearman correlation coefficients of miRNA-pairs - 5 -](#_Toc486956238)

[Comments to Supplementary Table S3 - 6 -](#_Toc486956239)

[**Supplementary Figure S2.**  Kaplan–Meier analyses - 7 -](#_Toc486956240)

[**Supplementary Table S4.** Target genes of miR-199-3p and miR-214-3p - 8 -](#_Toc486956241)

**Supplementary Table S1.** Details of the TaqMan microRNA assays ^1^

| **Assay name** | **Assay ID** | **miRBase accession no.** | **miRBase ID, version 21** | **Sequence** |
| --- | --- | --- | --- | --- |
| hsa-miR-100-5 | 000437 | MIMAT0000098 | hsa-miR-100-5p | AACCCGUAGAUCCGAACUUGUG |
| hsa-miR-101-3p | 002253 | MIMAT0000099 | hsa-miR-101-3p | UACAGUACUGUGAUAACUGAA |
| hsa-miR-125a-5p | 002198 | MIMAT0000443 | hsa-miR-125a-5p | UCCCUGAGACCCUUUAACCUGUA |
| hsa-miR-130b-3p | 000456 | MIMAT0000691 | hsa-miR-130b-3p | CAGUGCAAUGAUGAAAGGGCAU |
| hsa-miR-141-3p | 000463 | MIMAT0000432 | hsa-miR-141-3p | UAACACUGUCUGGUAAAGAUGG |
| hsa-miR-148b-3p | 000471 | MIMAT0000759 | hsa-miR-148-3p | UCAGUGCAUCACAGAACUUUGU |
| hsa-miR-151a-5p | 002642 | MIMAT0004697 | hsa-miR-151a-3p | UCGAGGAGCUCACAGUCUAGU |
| hsa-miR-199a-3p | 002304 | MIMAT0000232 | hsa-miR-199a-3p | ACAGUAGUCUGCACAUUGGUUA |
| hsa-miR-205-5p | 000509 | MIMAT0000266 | hsa-miR-205-5p | UCCUUCAUUCCACCGGAGUCUG |
| hsa-miR-214-3p | 002306 | MIMAT0000271 | hsa-miR-214-3p | ACAGCAGGCACAGACAGGCAGU |

^1^TaqMan microRNA Assays from Applied Biosystems for the examined mature miRNAs that are identified by the permanently assigned miRBase accession number, the miRBase-prescribed ID related to the miRBase version 21 (<http://www.mirbase.org>), and the nucleotide sequence.

**Supplementary Fig. S1.** Discriminative accuracy and capacity of miRNA models to differentiate between non-malignant bladder tissue (n=73) and muscle-invasive bladder cancer (MIBC) tissue samples (n=156). Model A (all six miRNAs) and Model B (three miRNAs after backward elimination) built by binary logistic regression were assessed by using (A) receiver-operating characteristics curve analysis and (B) decision curve analysis in comparison to miR-130b-3p as the best single discriminative miRNA indicator (Supplementary Table 2).

**Supplementary Table S2.** Receiver-characteristic curve analyses of single miRNAs and miRNA combinations to discriminate between non-malignant and MIBC tissue

| **miRNA** | **AUC**  **(95% CI)** | ***P*‑value different to AUC=0.5** | **Differentiating ability at the Youden index^1^** | | **Overall correct classifi-cation (%)** |
| --- | --- | --- | --- | --- | --- |
|  |  |  | **Sensitivity (95% CI)** | **Specificity (95% CI)** |  |
| Single miRNA |  |  |  |  |  |
| miR-100-5p | 0.79  (0.74–0.85) | <0.0001 | 61  (53–69) | 88  (78–94) | 70 |
| miR-130b-3p | 0.86  (0.80–0.91) | <0.0001 | 79  (71–85) | 81  (70–89) | 81 |
| miR-141-3p | 0.69  (0.62–0.76) | <0.0001 | 43  (41–58) | 81  (70–89) | 68 |
| miR-199a-3p | 0.77  (0.71–0.83) | <0.0001 | 58  (50–66) | 90  (81–96) | 71 |
| miR-205-5p | 0.54  (0.46–0.61) | 0.333 | 37  (29–45) | 82  (72–90) | 68 |
| miR-214-3p | 0.74  (0.68–0.81) | <0.0001 | 46  (38–54) | 95  (87–90) | 69 |
| Combined miRNAs |  |  |  |  |  |
| All miRNAs (Model A)^2^ | 0.90  (0.85–0.94) | <0.0001 | 77  (69–83) | 92  (83–97) | 85 |
| Optimized signature (Model B: miR‑100/‑130b/‑214)^3^ | 0.90  (0.84–0.94) | <0.0001 | 79  (71–85) | 88  (78–94) | 84 |
| Ratio of miR-100 to miR-130b | 0.89  (0.85–0.94) | <0.0001 | 81  (74-87) | 86  (76–93) | 82 |

AUC = area under the ROC curve; CI = confidence interval; MIBC = muscle-invasive bladder cancer.

^1^As cutoff for the evaluation of data was used the maximum value of the Youden index corresponding to the overall diagnostic effectiveness with the equal weight to sensitivity and specificity.

^2^Calculated by a combined model using binary logistic analysis with all six listed miRNAs.

^3^Calculated by a model build by binary logistic analysis using the backward elimination procedure (entry: *P* <0.05; removal: *P*  >0.10) with all six listed miRNAs resulting in a three-miRNA-signature.

**Comments to Supplementary Fig. S1 and Supplementary Table S2**

The differential expression of miRNAs as shown in Figure 1 implies their potential as discriminative tissue biomarkers. Receiver operating characteristic (ROC) analyses were performed to illustrate these characteristics. All miRNAs except for miR-205-5p revealed area under curve values indicating significant abilities to distinguish normal from malignant bladder tissue except for miR-205-5p (Supplementary Table 2). When including all miRNAs in binary logistic regression analyses (Model A), an overall correct classification of 85% was observed while the backward elimination approach resulted in a pattern of three miRNAs (Model B: miR-100-5p, miR-130b-3p, and miR-214-3p) with comparable sensitivities, specificities and correct classification rates. With miR-130b-3p alone or the ratio of miR-100-5p to miR-130b-3p, overall correct classifications of 81% and 82% were still achieved. However, the decision curve analysis proved equal curves for the reduced Model B with only three miRNAs in comparison to Model A including all six miRNAs, but distinctly higher benefit values than miR-130b-3p alone.

**Supplementary Table S3.** Spearman rank correlation coefficients between miRNA-pairs in non‑malignant and MIBC tumor tissue samples

| **miRNA pairs** | | **Spearman rank correlation coefficients (r_s_)** | | |
| --- | --- | --- | --- | --- |
|  | | All samples  (n = 229) | Non-malignant  (n = 73) | MIBC  (n = 156) |
| **miR-100-5p** | miR-130b-3p | -0.295 ^a^ | 0.261 ^a,b^ | -0.109 ^b^ |
|  | miR-141-3p | -0.404 ^a^ | -0.481^a^ | -0.248 ^a^ |
|  | miR-199a-3p | 0.382 ^a^ | 0.283 ^a^ | 0.265 ^a^ |
|  | miR-205-5p | -0.143 ^a^ | -0.456 ^a,b^ | 0.032 ^b^ |
|  | miR-214-3p | 0.420 ^a^ | 0.569 ^a,b^ | 0.272 ^a,b^ |
| **miR-130b-3p** | miR-100-5p | -0.295 ^a^ | 0.261 ^a,b^ | -0.109 ^b^ |
|  | miR-141-3p | 0.346 ^a^ | 0.190 | 0.251 ^a^ |
|  | miR-199a-3p | -0.379 ^a^ | -0.155 | -0.178 ^a^ |
|  | miR-205-5p | 0.047 | 0.132 | -0.029 |
|  | miR-214-3p | -0.329 ^a^ | 0.177 ^b^ | -0.205 ^a,b^ |
| **miR-141-3p** | miR-100-5p | -0.404 ^a^ | -0.481 ^a^ | -0.248 ^a^ |
|  | miR-130b-3p | 0.346 ^a^ | 0.190 | 0.251 ^a^ |
|  | miR-199a-3p | -0.334 ^a^ | -0.359 ^a^ | -0.206 ^a^ |
|  | miR-205-5p | 0.550 ^a^ | 0.888^a,b^ | 0.464 ^a,b^ |
|  | miR-214-3p | -0.379 ^a^ | -0.333 ^a^ | -0.285 ^a^ |
| **miR-199a-3p** | miR-100-5p | 0.382 ^a^ | 0.283 ^a^ | 0.265 ^a^ |
|  | miR-130b-3p | -0.379 ^a^ | -0.155 | -0.178 ^a^ |
|  | miR-141-3p | -0.334 ^a^ | -0.359 ^a^ | -0.206 ^a^ |
|  | miR-205-5p | -0.076 | -0.255 ^a^ | -0.006 |
|  | miR-214-3p | 0.891 ^a^ | 0.617 ^a,b^ | 0.939 ^a,b^ |
| **miR-205-5p** | miR-100-5p | -0.143 ^a^ | -0.456 ^a,b^ | -0.032 ^b^ |
|  | miR-130b-3p | 0.047 | 0.132 | -0.029 |
|  | miR-141-3p | 0.550 ^a^ | 0.888 ^a,b^ | 0.464 ^a,b^ |
|  | miR-199a-3p | -0.076 | -0.255^a^ | -0.006 |
|  | miR-214-3p | -0.118 | -0.213 | 0.064 |
| **miR-214-3p** | miR-100-5p | 0.420 ^a^ | 0.569 ^a,b^ | 0.272 ^a,b^ |
|  | miR-130b-3p | -0.329 ^a^ | 0.177 ^b^ | -0.205 ^a,b^ |
|  | miR-141-3p | -0.379 ^a^ | -0.333 ^a^ | -0.285 ^a^ |
|  | miR-199a-3p | 0.891 ^a^ | 0.617^a,b^ | 0.939 ^a,b^ |
|  | miR-205-5p | -0.118 | -0.213 | -0.064 |

MIBC = muscle-invasive bladder cancer.

^a^Significant correlation coefficients of at least *P* <0.05 between the indicated miRNA pairs in the respective group of tissue samples.

^b^Significantly different correlation coefficients of the indicated miRNA pairs of at least *P* <0.05 between the non-malignant and MIBC tissue samples.

**Comments to Supplementary Table S3**

Supplementary Table 3 summarizes the correlation cofficients between the various miRNAs depending on the kind of tissue samples. Out of the possible 15 miRNA pairs, ten pairs correlated with each other in the respective tissue sample but only seven pairs were similarly correlated in non-malignant and malignant tissue samples. However, different correlation coefficients–either with regard to their level or clearly inversely–were observed between non-malignant and MIBC tissue for six miRNA pairs, namely, miR-100-5p with miR-130b-3p, miR-205-5p and miR-214-3p; miR-130b-3p with miR-214-3p; miR-141-3p with miR-205-5p, and miR-199a-3p with miR-214-3p. These correlation differences reflect the differently increased or decreased expression in the tissue samples as described above.

**Supplementary Fig. S2.** Kaplan–Meier analyses of overall survivals of muscle-invasive bladder cancer patients after radical cystectomy in association to clinicopathological variables. The curves from the whole cohort of 156 patients listed in Table 1 are presented according to (A) age, (B) sex, (C) pT status, (D) histological grade, (E) metastatic lymph node status, and (F) adjuvant chemotherapy. The log-rank test was used for evaluating statistically significant differences between the survival probabilities.

| **Supplementary Table S4.** Target genes of miR-199-3p and miR-214-3p^1^ | | | | |
| --- | --- | --- | --- | --- |
| **Gene name** | **Description** | **Location** | **Aliases** | **Ref./cancer/miR** |
| ARL2 | ADP ribosylation factor like GTPase 2 [*Homo sapiens* (human)] | Chromosome 11, NC_000011.10 (65014113..65022185) | ARFL2 | [48] cervical cancer miR-214 |
| AURKA | aurora kinase A [*Homo sapiens* (human)] | Chromosome 20, NC_000020.11 (56369389..56392337, complement) | AIK, ARK1, AURA, BTAK, PPP1R47, STK15, STK6, STK7 | [39] prostate cancer miR-199a |
| DNMT1 | DNA methyltransferase 1 [*Homo sapiens* (human)] | Chromosome 19, NC_000019.10 (10133344..10195135, complement) | ADCADN, AIM, CXXC9, DNMT, HSN1E, MCMT, m.HsaI | [52] testicular cancer miR-199a/-214 |
| FLT1 | fms related tyrosine kinase 1 [*Homo sapiens* (human)] | Chromosome 13, NC_000013.11 (28300346..28495128, complement) | FLT, FLT-1, VEGFR-1, VEGFR1 | [43] hepatocellular carcinoma miR-199a |
| GSK3B | glycogen synthase kinase 3 beta [*Homo sapiens* (human)] | Chromosome 3, NC_000003.12 (119821321..120094417, complement) |  | [37] kidney cancer miR-199a |
| HGF | hepatocyte growth factor [*Homo sapiens* (human)] | Chromosome 7, NC_000007.14 (81699006..81770438, complement) | DFNB39, F-TCFB, HPTA, SF, HGF | [43] hepatocellular carcinoma miR-199a |
| ITGA3 | integrin subunit alpha 3 [*Homo sapiens* (human)] | Chromosome 17, NC_000017.11 (50055968..50090485) | CD49C, FRP-2, GAP-B3, GAPB3, ILNEB, MSK18, VCA-2, VL3A, VLA3a | [42] bladder cancer miR-199a |
| KDR | kinase insert domain receptor [*Homo sapiens* (human)] | Chromosome 4, NC_000004.12 (55078259..55125595, complement) | CD309, FLK1, VEGFR, VEGFR2 | [43] hepatocellular carcinoma miR-199a |
| LNC2 | lipocalin 2 [*Homo sapiens* (human)] | Chromosome 9, NC_000009.12 (128149430..128153455) | 24p3, MSFI, NGAL, p25 | [50] bladder cancer miR-214 |
| MMP2 | matrix metallopeptidase 2 [*Homo sapiens* (human)] | Chromosome 16, NC_000016.10 (55478830..55506691) | CLG4, CLG4A, MMP-2, MMP-II, MONA, TBE-1 | [43] hepatocellular carcinoma miR-199a |
| MMP9 | matrix metallopeptidase 9 [Homo sapiens (human)] | Chromosome 20, NC_000020.11 (46008908..46016561) | CLG4B, GELB, MANDP2, MMP-9 | Falzone, 2016 683 /id} bladder cancer miR-214 |
| PDRG1 | p53 and DNA damage regulated 1 [*Homo sapiens* (human)] | Chromosome 20, NC_000020.11 (31944955..31952080, complement) | C20orf126, PDRG | [33] bladder cancer miR-214 |
| PSMD10 | proteasome 26S subunit, non-ATPase 10 [*Homo sapiens* (human)] | Chromosome X, NC_000023.11 (108084205..108091644, complement) | dJ889N15.2, p28, p28(GANK) | [52] testicular cancer miR-199a/-214 |
| RFWD2 | ring finger and WD repeat domain 2 [*Homo sapiens* (human)] | Chromosome 1, NC_000001.11 (175944826..176207279, complement) | COP1, RNF200 | [47] breast cancer miR-214 |
| SLC34A2 | solute carrier family 34 member 2 [*Homo sapiens* (human)] | Chromosome 4, NC_000004.12 (25655813..25678748) | NAPI-3B, NAPI-IIb, NPTIIb | [51] bladder cancer miR-214 |
| TP53 | tumor protein p53 [*Homo sapiens* (human)] | Chromosome 17, NC_000017.11 (7668402..7687550, complement) | BCC7, LFS1, P53, TRP53 | [52] testicular cancer miR-199a/-214 |
| VEGFA | vascular endothelial growth factor A [*Homo sapiens* (human)] | Chromosome 6, NC_000006.12 (43770209..43786487) | MVCD1, VEGF, VPF | [43] hepatocellular carcinoma miR-199a |

^1^The data of the target genes were taken from the NCBI data base "Gene". The references in the last column refer to the references in the main text.
